# Deep Neural Networks and Transfer Learning on a Multivariate Physiological Signal Dataset

**DOI:** 10.3390/bioengineering8030035

**Published:** 2021-03-06

**Authors:** Andrea Bizzego, Giulio Gabrieli, Gianluca Esposito

**Affiliations:** 1 Department of Psychology and Cognitive Science, University of Trento, 38068 Rovereto (Trento), Italy; andrea.bizzego@unitn.it; 2 Psychology Program, School of Social Sciences, Nanyang Technological University, Singapore 639798, Singapore; GIULIO001@e.ntu.edu.sg; 3 Lee Kong Chian School of Medicine, Nanyang Technological University, Singapore 639798, Singapore

**Keywords:** multivariate data, physiological signals, signal processing, artificial intelligence, deep neural networks, transfer learning

## Abstract

While Deep Neural Networks (DNNs) and Transfer Learning (TL) have greatly contributed to several medical and clinical disciplines, the application to multivariate physiological datasets is still limited. Current examples mainly focus on one physiological signal and can only utilise applications that are customised for that specific measure, thus it limits the possibility of transferring the trained DNN to other domains. In this study, we composed a dataset (n=813) of six different types of physiological signals (Electrocardiogram, Electrodermal activity, Electromyogram, Photoplethysmogram, Respiration and Acceleration). Signals were collected from 232 subjects using four different acquisition devices. We used a DNN to classify the type of physiological signal and to demonstrate how the TL approach allows the exploitation of the efficiency of DNNs in other domains. After the DNN was trained to optimally classify the type of signal, the features that were automatically extracted by the DNN were used to classify the type of device used for the acquisition using a Support Vector Machine. The dataset, the code and the trained parameters of the DNN are made publicly available to encourage the adoption of DNN and TL in applications with multivariate physiological signals.

## 1. Introduction

In medicine and other medical sciences, physiological recordings are widely employed to monitor and assess the health status of patients [1,2]. Since physiological signals are directly controlled by the Autonomic Nervous System, they are also less influenced by social inferences and explicit behavioral responses [3]. The analysis of multivariate physiological signals allows the investigation of psycho-physiological regulatory mechanisms of the individual from different perspectives and enables researchers to study how humans react to external stimuli and adapt to environmental changes [2,4,5].

However, employing multivariate physiological signals in the research process comes at a cost: first, physiological measurements are highly sensitive to the presence of noise and artifacts; second: each type of physiological signal requires specialized hardware and tools for data collection and processing. For these reasons, the typical approach to multivariate signals analysis involves some steps of visual inspection to assess the quality of the recordings (e.g., to discriminate between usable and unusable samples), and select the most appropriate tools and procedures for analysis [6]. In an attempt to reduce the subjectivity of these steps, Artificial Intelligence (AI) methods have been recently introduced. AI was employed to automatize the phases of preprocessing and analysis of recorded signals, therefore increasing the reproducibility and reducing the likelihood of human errors. In Gabrieli et al. [6], different Machine Learning models proved to be able to efficiently discriminate between usable and unusable samples in multiple infants’ fixation time studies, while numerous studies employed AI models on preprocessed physiological signals, for instance, to identify ventricular hypertrophy [7], arrhythmia [8], muscle fatigue [9], and stress [10].

One of the most promising AI methods is the use of Deep Neural Networks (DNN) [11,12]. DNNs are a family of Machine Learning methods that rely on the use of modular architectures, based on multiple nonlinear processing units (layers), to extract high-level patterns from data. Thanks to the hierarchical structure of the layers, DNN progressively obtains high-level features from low-level representations [13], thus transforming input data into a multi-dimensional representation useful to solve the classification task [14]. However, optimally training DNN typically requires a much larger amount of data than statistical Machine Learning models: the Transfer Learning (TL) approach overcomes this issue partially. The key idea of TL is leveraging on DNN which were already trained on different domains or datasets, and adapting them to the new task, by partially re-trained using data from the new task [15]. Thanks to the availability of large-scale data and pre-trained architectures [16,17], the TL approach boosted the adoption of DNN in several disciplines and out-performed previous state-of-art Machine Learning models and, in some cases, human expertise [18].

DNNs based on Convolutional Neural Networks (CNN) are currently the state-of-the-art models in several image classification applications that adopt an “end-to-end” paradigm: i.e., images are directly processed by the CNN without prior processing (e.g., feature extraction). The adoption of DNN and CNN in applications based on medical data (bio images and physiological signals) is rapidly growing, with a wide range of applications [19,20,21,22,23].

DNN and CNN have also been applied to physiological signals. For instance, a DNN, was successfully employed to design a biometric identification signal based on Electrocardiogram (ECG) [24], or to identifyventricular and supraventricular ectopic beats [25]. In a work by Xu et al. [26], a DNN was effectively employed to classify the type of heartbeat patterns (e.g., normal beat, arrhythmia) from raw ECG. Similar procedures were employed on other physiological signals: Yu and Sun [27] used a DNN to classify emotions from phasic and tonic components of the Electrodermal Activity (EDA), while Mukhopadhyay and Samui [14] employed DNN to classify limbic movements from Electro-myogram (EMG) signals. A recent review by Rim et al. [28], surveyed the application of DNN on four different Physiological signals (EMG, ECG, Electro-oculogram, and Electro-encephalogram) in both single- and multivariate datasets. The datasets were drawn from both public and private repositories and were identified based on the methodology employed to analyze the data. Inclusion criteria for being reviewed included the employment of DNN for clinical applications and emotion recognition. The use of DNN on physiological signals can effectively and efficiently support clinicians in their work, for example by identifying heart diseases [29,30] or brain disease [31,32,33]. However, the majority of DNN employed in physiological signals analysis target only a specific type of physiological signal. As a consequence, they are not appropriate for analyzing multivariate datasets, which are rapidly becoming the standard in medicine and neuroscience [34], and are application-specific, meaning trained models cannot be easily used for TL [35].

### Aim of This Study

The aim of this study is to obtain a DNN to classify multivariate data and extract physiological patterns. From the clinical perspective, we aim at setting the base for the development of computer-assisted diagnostics based on physiological signals, to keep pace with other clinical disciplines based on imaging.

This study provides three main practical contributions to facilitate the adoption of DNNs for multivariate physiological signals: (i) We apply a DNN to classify six types of physiological signals: Electrocardiogram, Electrodermal activity, Electromyogram, Photoplethysmogram, Respiration, and Acceleration. Signals were composed into a dataset of 813 physiological signals collected from 232 subjects using four different acquisition devices. (ii) We test the efficacy of the TL approach. Based on high-level features extracted from the trained DNN, we applied a standard Machine Learning method to classify the type of device. Although standard TL approaches based on re-training of weights might be more effective, our example demonstrates that the DNN extracts signal patterns that can be ported to other domains. (iii) We make available the training dataset as well as the DNN architecture with the pre-trained weights, with the aim of encouraging and facilitating the adoption of DNN with multivariate physiological signal datasets.

## 2. Materials and Methods

### 2.1. Peripheral Physiological Signals

In this study, we consider six peripheral physiological signals that are commonly used in multivariate experimental settings.

The Electrocardiogram (ECG) and Photo-plethysmogram (PPG) are both recorded to measure the cardiac activity. ECG employs surface electrodes to detect the electrical potential of cardiac cells, PPG is based on optical sensors, and it measures blood volume changes in the microvascular bed of tissue [36]. Both signals can be used to investigate the Heart Rate Variability: how the heart rate changes in response to physiological needs and external stimuli, under the control of the Autonomic Nervous System. EDA is the measurement of changes in the electrical properties of the skin that occur in response to sweat secretion. Since the sweat glands are under the control of the sympathetic nervous system, EDA is used to investigate emotional arousal [37,38]. EMG is the study of the muscles’ electrical signals, which are generated by the contraction and relaxation of muscles. Analysis of the EMG is used for both clinical and research purposes since it can be used in biomechanics, motor, and neuromuscular physiology, and emotion recognition [39,40,41]. Information about individuals’ respiration patterns can provide researchers with details about the status of both the central and peripheral nervous system [42]. A respiratory signal (RESP) can be recorded using an elastic band positioned around an individual’s chest to measure the relative volumetric expansion. Finally, we consider the acceleration signals (ACC) used to detect body movements which are used in clinical settings, not only to study conditions associated with impaired movement but also stereotypical motor movements in children with autism spectrum disorders [43,44,45].

### 2.2. Multivariate Signals’ Datasets

Physiological signals used in this study were obtained from four publicly available datasets of multivariate signals: the Database for Emotion Analysis using Physiological signals (DEAP, reference [46], available at http://www.eecs.qmul.ac.uk/mmv/datasets/deap/, accessed on 5 March 2021), the Wearable and Clinical Signals dataset (WCS, reference [47], available at https://doi.org/10.21979/N9/42BBFA, accessed on 5 March 2021), a dataset used to investigate Synchrony In Dyads (SID, reference [48], available at https://doi.org/10.21979/N9/O9ADTR, accessed on 5 March 2021), and a dataset used to investigate the Perception of Implicit Aesthetic Pleasure (PIAP, reference [49], available at https://doi.org/10.21979/N9/YCDXNE, accessed on 5 march 2021).

The DEAP dataset was created to investigate the association between physiological response and emotional stimuli and provide data for Machine Learning applications aimed at emotion recognition. From the list of peripheral signals included in the DEAP, we selected the EDA and the PPG. All signals were collected with the same sampling rate of 512 Hz using the Biosemi ActiveTwo acquisition unit. The DEAP dataset provides signals of 32 subjects.

The WCS dataset was created to compare signals collected with clinical-grade and wearable devices and to test and validate algorithms to assess the quality of the data. Specifically, in this study, we consider five different types of signals, collected with 2 different devices: (i) the FlexComp acquisition unit, operating at a sampling rate of 2048 Hz, used to collect four physiological signals: ECG, EDA, PPG, and RESP; and (ii) the Empatica E4, a wrist-band used to collect PPG (64 Hz), EDA (4 Hz), and ACC (32 Hz). The WCS dataset provides signals of 18 subjects in two experimental conditions: at rest (*n* = 18) and while performing an in-place walking (*n* = 18).

The SID dataset was used to investigate the physiological synchrony between members of dyads with different levels of relationship. We considered three types of physiological signals (ECG, EDA, EMG) provided by the SID dataset, all collected with the FlexComp acquisition unit, operating at a sampling rate of 2048 Hz. The SID dataset provides signals of 124 subjects (62 dyads).

The PIAP dataset was used to investigate the perceived aesthetics of web-pages and images and it contains several physiological signals of young adults (N=59, 33 females, Mean age = 21.52 years) while they were asked to assess the aesthetic appeal of websites and of emotional pictures. Specifically, for this dataset, we consider three types of physiological signals: ECG, EDA, EMG (*corrugator supercilii*), recorded with a sampling rate of 1000 Hz using an open-source biosignal acquisition platform [50]. In this study, a subset of 22 participants has been selected.

Overall (Table 1), for this study, we composed a dataset with 6 types of signals from 4 acquisition devices and 232 subjects, totaling 813 samples of physiological signals. The dataset was divided into two partitions, preserving the percentage of samples for each class. The first partition (n = 609, 75% of the dataset) was used for training the AI models, the second partition (n = 204, remaining 25% of the dataset) was uniquely used for testing. Using separate partitions for training and testing AI models is a common practice to ensure unbiased evaluations of the predictive performances.

### 2.3. Pre-Processing

To reduce the memory size of the dataset, the original signals were cropped into segments with a maximum length of 300 s. The selected segment corresponded to the first 300 s when the signal was shorter than 600 s; otherwise, to the portion between 300 to 600 s. This was to avoid the initial portions of the signal, where noise and artifacts are more frequent because the preparation of the subject or the experiment is still ongoing.

In line with the end-to-end approach, only two pre-processing steps were performed: normalization and resampling. Both steps were required to comply with the DNN architecture. The normalization was necessary to uniform the range of the input signal: all signals were standardized by subtracting the mean value and dividing by the standard deviation. The resampling was performed to overcome the heterogeneity of sample rates of the different signal acquisition devices. Signals were resampled with a resampling rate of 100 Hz, which was empirically selected as a trade-off: while higher sampling rates would allow for more informative signals, lower sampling rates would increase the speed of the training and processing.

A key step to improve the performance of DNN is data augmentation, the procedure of enriching an existing dataset, by randomly modifying its datapoints. Data augmentation prevents the network from relying on instance-specific patterns, thus preventing overfitting and improving generalizability [51]. In our implementation, every time a signal is used to train the network, we randomly select the 10 s length portion of the signal that is actually used as input to the DNN. When used to test the network performance, the central 10 s length portion is selected instead.

### 2.4. Deep Learning Architecture

**Figure 1:**
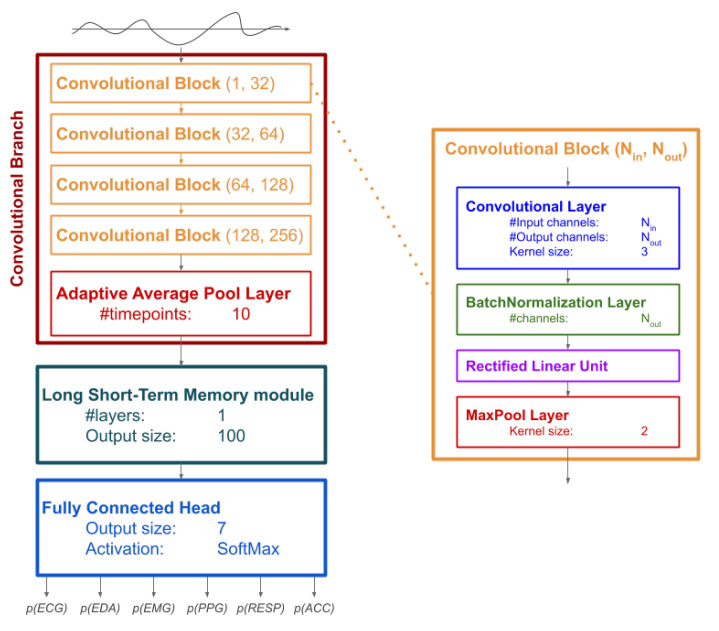
Diagram representing the architecture of the Deep Neural Network (DNN) used in this study. Left: the complete network with the three components (the Convolutional Branch, the Long Short-Term Memory module, and the Fully Connected Head) and parameters used for each layer. The input signal is processed by the DNN to output the probability of belonging to each of the six signal types considered in the study. Right: structure of a general Convolutional Block with $N_{in}$ input channels $N_{out}$ output channels.

The DNN architecture used in this study has three sequential components (Figure 1): (i) a convolutional branch; (ii) a Long Short-Term Memory (LSTM) module; a Fully Connected Head (FCH).

The convolutional branch is composed of 4 convolutional blocks, each composed of a convolutional layer (with kernel size set to 3), a normalization layer (using batch normalization [52]), a Rectified Linear Unit [53], and a pooling layer based on maximum (with kernel size set to 2). The convolutional layer of each block expands the number of channels: the first expands from 1 to 32 channels, the second to fourth blocks duplicate the number of channels up to 256 channels for the fourth block. The structure of the convolutional branch is directly inspired by Convolutional Neural Networks that are largely used on images and videos. The main difference is that, in our study, we use a one-dimensional, layer—which is more appropriate to process signals—instead of two-dimensional layers which are used for images. After the 4 convolutional blocks, an additional pooling layer is used to compute the average of the convoluted (multichannel) signal at 10-time points, thus obtaining a temporal sequence of 10 elements, each with 256 values (one for each channel).

The LSTM module [54,55] is a recursive layer that is used in Neural Networks to leverage the specific properties of sequential data. In the implementation used in this study, the LSTM module has an output size set to 100 and a single layer. The LSTM processes the output of the convolutional layer one element at a time: each time, the output of the LSTM is updated considering the new element in the sequence and the output obtained from the previous element. The output of the LSTM module is a vector with 100 elements, which is the result of the recursion on the last element of the sequence.

The final component of the DNN is the FCH, which linearly combines the 100 elements from the LSTM module to output a vector of 7 elements, on which a Softmax is finally applied to compute the probability of belonging to any of the 6 types of signals.

The first two components (the convolutional branch and the LSTM module) are used as automated feature extractors in the TL experiment. Each signal is sequentially processed by both components, and the vector with 100 elements resulting from the LSTM module is the vector of the extracted features.

### 2.5. Analytic Plan

This study is composed of two separate Machine Learning experiments: the first focuses on training the DNN to classify the type of signals, the second uses the trained DNN as a feature extractor and uses a Support Vector Machine (SVM) to classify the type of device used to collect the signals. Both experiments are based on the same dataset and use the train partition to train the model, and the test partition to assess the performance of the trained model.

In the first experiment, the signals in the train partition are used to optimize the parameters of the DNN. The DNN adopts an end-to-end pipeline: the signals are directly processed by the sequence of the three components to output the probability that the signal belongs to each type of signal. The type with higher probability is considered the result of the classification. The training was iterated for 400 epochs; in each epoch, all the signals in the training dataset are used, randomly divided into batches of 32 signals. The DNN parameters are optimized to minimize the Cross-Entropy Loss [56] between the true and predicted types of signal. For the optimization of the DNN parameters, we used the Adam optimizer [57], with an initial learning rate of 0.001 which was divided by 10 every 50 epochs. The performance of the DNN was evaluated using the multi-class Matthew Correlation Coefficient (mMCC) and the Confusion Matrix for both the train and test partitions [58].

In the second experiment, we provide a demonstrative example of how the previously trained DNN can be useful for TL approaches. TL relies on the fact that many of the features that are automatically extracted by the DNN can be useful for other applications. All signals in the dataset were then processed by the convolutional branch and LSTM module to obtain 100 features for each signal. These features, which are expected to synthesize patterns useful to classify the type of signal, are transferred to classify the type of device that was used to collect the signal. A SVM model, with linear kernel and regularization parameter C=1, was trained on the features extracted from the signals of the train partition and evaluated on features from signals of the test partition. The performance of the SVM was evaluated using the mMCC and the Confusion Matrix for both the train and test partitions [58].

The implementation of the mMCC and SVM models and training of the SVM model were based on the scikit-learn Python package (v0.23.2, reference [59]). The implementation of the DNN and training methods, including the loss function and optimizer, were based on pytorch (v1.3.1, reference [60]).

### 2.6. Code and Data Availability

The code, the dataset with pre-processed signals, and the parameters of the trained DNN are available at https://gitlab.com/abp-san-public/dl-signal-classification (accessed on 5 march 2021).

## 3. Results

**Figure 2:**
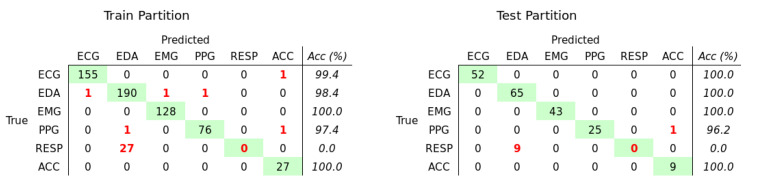
Confusion Matrices that show the result of the classification of the type of signal, on the train (**left**) and test (**right**) partitions. On the diagonal (green), the numbers of correctly classified samples for each type of signal. Out of the diagonal, in red, the numbers of mis-classified samples.

The network trained to classify the type of signal achieved a mMCC of 0.931 on the train partition and 0.938 on the test partition. The confusion matrices (Figure 2) showed a high class accuracy (>96.2%) for all types of signals, except RESP.

Overall, the reported performances were similar in both the train and test partitions, suggesting that the training process was able to efficiently detect and extract characterizing patterns and avoid over-fitting. However, we observed that the model was not able to correctly classify the RESP signals, which were confounded with EDA.

**Figure 3:**
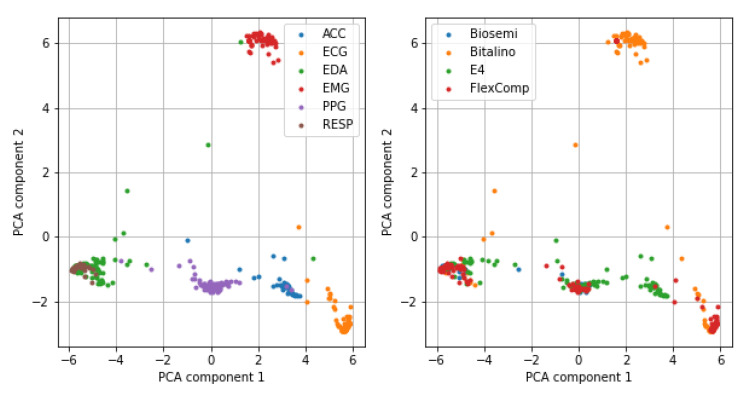
Representation of the input samples in the two-dimensional space defined by the Principal Component Analysis (PCA) of the extracted features. **Left**: colored by type of signal; **Right**: colored by type of device.

The first two components of the trained network were used to extract the features for the classification of the type of device. To visualize the results of the feature extraction, we apply a dimensionality reduction on the extracted features, based on two-dimensional Principal Component Analysis (PCA). We observed (Figure 3) that the features generated by DNN efficiently separate the type of signals; however, we also recognized the lack of separation between RESP and EDA signals, which motivates the low-class accuracy for the RESP type.

**Figure 4:**
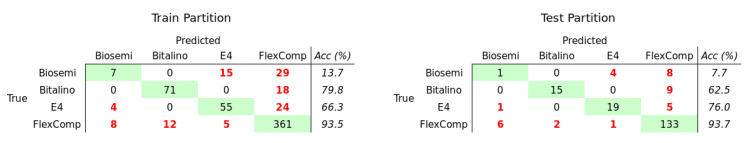
Confusion Matrices that show the result of the classification of the type of device, on the train (**left**) and test (**right**) partitions. On the diagonal (green), the numbers of correctly classified samples for each type of device. Out of the diagonal, in red, the numbers of mis-classified samples.

The SVM model, trained on the extracted features to predict the type of device, achieved a mMCC of 0.638 on the train partition and of 0.609 on the test partition; the confusion matrices reported similar performances in both partitions (Figure 4).

The device with the lowest class accuracy was the Biosemi (13.7% on train, 7.7% on test), while the most frequent classification error was attributing a signal to the FlexComp device. Again, the motivation can be found by observing the results of the PCA analysis (Figure 3): since the same device is used to collect multiple types of signals, the extracted features, which well separated the types of signals, are not as optimal to discriminate the devices.

## 4. Discussion

In this study, we demonstrated the use of DNN in the classification of multivariate physiological data. In particular, a key aspect of our study is that it focuses on six different types of physiological signals, instead of a limited number of physiological signals like similar studies have done [61,62,63].

We successfully employed DNN to classify the type of physiological signal, with a very good overall performance, demonstrated by the high mMCC on both the train and test partitions. Only one type of signal, the respiratory signal (RESP), was incorrectly classified, possibly due to the low number of samples available in the dataset. Future studies should focus on having a richer and more balanced dataset. Notably, the Convolutional branch and the LSTM components automatically learned to synthesize features that encode the relevant patterns to allow for discriminating the type of signals, as shown by the results of the PCA analysis.

Moreover, we provided a proof of concept to show how multivariate analysis can benefit from the TL approach, enabled by the use of DNN. The trained DNN was used to obtain generalized features of the signals: although created to encode signal patterns relevant to discriminate the type of signals, the extracted features were also useful to classify the type of device. Specifically, we adopted one of the simplest TL approaches: the extracted features were used to train a SVM model, achieving an overall mMCC of 0.638 on the train partition and of 0.609 on the test partition. We did not investigate how the achieved performance depends on the partitioning of signal types into the different devices in the original dataset; as well as it was out of the scope of this study to improve the performance of the TL task. We posit that, by adopting more specialized TL approaches, for instance, re-training only the FCH component, will allow better performance. The ability to classify the type of device itself may not be of immediate use. Instead, our TL implementation aimed at providing an example of how the approach facilitates the adoption of DNN in different and transversal applications based on multivariate data.

Drawbacks of using TL should be considered as well, especially when applied to biological or physiological data. If features learned by DNN usually outperform hand-crafted features, they are usually harder to interpret in biological terms [64,65,66,67]. When the emphasis on interpretability is a key factor, the use of TL and DNN, in general, should be paired with more standard approaches.

To be efficiently transferred to other domains, DNN should extract generic patterns. This is the reason we believe that existing examples of DNN applied to physiological signals, being trained on specialized tasks, would not allow transfer learning. Our DNN, being trained on a more generic task and on a multivariate dataset, would a better candidate to be transferred to other domains. However, more studies are needed to clarify this aspect: in the literature, only a few examples made use of DNN on multivariate physiological signals, and even fewer adopt TL.

Finally, the DNN can be easily improved by adding more physiological signals from other databases and by considering new types of signals, for instance, brain signals such as from Electro-encephalogram [46] and functional Near Infra-Red Spectroscopy [68,69].

### 4.1. Limitations

This study has some methodological issues, motivated by constraints and limitations due to the exploratory nature of the study and characteristics of the dataset.

First, in the preprocessing steps, we resampled all signals to a sampling rate of 100 Hz. While for some signals this rate is well above the one required to obtain significant physiological information (e.g., RESP, EDA), for other signals (e.g., ECG, EMG), this likely caused information losses. However, since the purpose of the network was to classify the type of signal, not to extract clinical or physiological evidence, we deem that the potential loss of information is acceptable, considering the gain in terms of increased processing speed. However, approaches targeting diagnostic tasks should consider higher sampling rates.

Second, we demonstrated the TL approach on the same dataset used for the main DNN, to reduce the overall complexity of the study. In reality, TL applications usually consider not only a different task or target but also different datasets.

Third, compared to networks used for medical images, the neural network used in this study is not very *deep*: it is composed of 20 layers (including normalization and activation layers), for a total number of 274,366 parameters. As a reference, the VGG-16 network [70] contains 39 layers, for a total of 138.4 M parameters. Using a relatively low number of layers was motivated by the fact of having a low number of samples (VGG-16 was originally trained on a dataset with 1.3 M images). If required in future applications, our architecture can be properly improved, provided that a dataset with an adequate sample size is used.

### 4.2. Implications

With a specific focus on multivariate physiological signals, the DNN approach adopted in this study can be easily adapted to simultaneously process multiple types of signals: convolutional branches can be used to extract a set of features from each type of signal that are then merged by the FCH [19].

The trained DNN has its immediate application with novel signal processing platforms (e.g., https://datagrok.ai, accessed on 5 March 2021), where the recognition of the type of signal is the key step to initiate or recommend the correct processing procedure.

In turn, we highlight the importance of sharing the weights of trained networks and benchmark datasets; these two practices have largely contributed to the adoption of DNN in other disciplines. However, compared to other medical fields (e.g., Nuclear Medicine, Histology and Pathology, Microscopy), the use of DNN on multivariate physiological signals is still quite unexplored. This is probably due to the lack of extended datasets and paucity of foundational applications, which, as in the case of medical imaging, could then be adapted to the specific use cases and improved. By providing a benchmark dataset and a working generalized DNN, we aim at providing a significant contribution to the development and diffusion of DNN for multivariate physiological signals.

The use of multivariate data in clinical applications mostly pertains to diagnostic and monitoring tasks (for instance in Intensive Care Units), where the efficacy of AI approaches can be fully exploited. It should be noted, however, that, to be reliably applied in real-time clinical applications, AI models require a delicate and demanding phase of training, where the availability of good datasets and computing resources are the key. The availability of general datasets and pre-trained models that can be adapted to more specific tasks using TL represents therefore a fundamental resource. Regarding the dataset used in this study, having different tasks and subjects from different populations is a key feature of our approach, since it allows for obtaining a more general model. In the case of clinical applications, this model can be further improved using a specialized dataset with subjects from the same population while executing the same task. While we believe the dataset and the pre-trained model developed in this study represent a key contribution to the adoption of AI for clinical applications, further studies are required to provide reliable guidelines and examples about how AI and TL should be efficiently applied to achieve reliable results.

## Figures and Tables

**Table 1:** Number of samples per type of signal and source dataset, with sampling frequency (in Hz) and devices.

Dataset	ECG	EDA	EMG	PPG	RESP	ACC	N. of Samples	Device
DEAP	-	32 (512 Hz)	-	32 (512 Hz)	-	-	64	Biosemi
WCS	36 (2048 Hz)	36 (2048 Hz)	-	36 (2048 Hz)	36 (2048 Hz)	-	144	Flexcomp
	-	36 (4 Hz)	-	36 (64 Hz)	-	36 (32 Hz)	108	E4
SID	128 (2048 Hz)	128 (2048 Hz)	128 (2048 Hz)	-	-	-	384	Flexcomp
PIAP	44 (1000 Hz)	26 (1000 Hz)	43 (1000 Hz)	-	-	-	113	Bitalino
Total	208	258	171	104	36	36	813	

## Data Availability

All the data used in this study were obtained from publicly available datasets: the Database for Emotion Analysis using Physiological signals (DEAP, reference [46], available at http://www.eecs.qmul.ac.uk/mmv/datasets/deap/, accessed on 5 March 2021), the Wearable and Clinical Signals dataset (WCS, reference [47], available at https://doi.org/10.21979/N9/42BBFA, accessed on 5 March 2021), a dataset used to investigate Synchrony In Dyads (SID, reference [48], available at https://doi.org/10.21979/N9/O9ADTR, accessed on 5 March 2021), and a dataset used to investigate the Perception of Implicit Aesthetic Pleasure (PIAP, reference [49], available at available at https://doi.org/10.21979/N9/YCDXNE, accessed on 5 March 2021).

## References

[1] Bizzego A., Battisti A., Gabrieli G., Esposito G., Furlanello C. (2019). pyphysio: A physiological signal processing library for data science approaches in physiology. SoftwareX.

[2] Wagner J., Kim J., André E. From physiological signals to emotions: Implementing and comparing selected methods for feature extraction and classification. Proceedings of the IEEE International Conference on Multimedia and Expo.

[3] Gabrieli G., Azhari A., Esposito G. (2020). PySiology: A python package for physiological feature extraction. Neural Approaches to Dynamics of Signal Exchanges.

[4] Kreibig S.D. (2010). Autonomic nervous system activity in emotion: A review. Biol. Psychol..

[5] Levenson R.W. (2014). The autonomic nervous system and emotion. Emot. Rev..

[6] Gabrieli G., Balagtas J.P.M., Esposito G., Setoh P. (2020). A Machine Learning approach for the automatic estimation of fixation-time data signals’ quality. Sensors.

[7] Jothiramalingam R., Jude A., Patan R., Ramachandran M., Duraisamy J.H., Gandomi A.H. (2020). Machine learning-based left ventricular hypertrophy detection using multi-lead ECG signal. Neural Comput. Appl..

[8] Bulbul H.I., Usta N., Yildiz M. Classification of ECG arrhythmia with machine learning techniques. Proceedings of the 16th IEEE International Conference on Machine Learning and Applications (ICMLA).

[9] Karthick P., Ghosh D.M., Ramakrishnan S. (2018). Surface electromyography based muscle fatigue detection using high-resolution time-frequency methods and machine learning algorithms. Comput. Methods Programs Biomed..

[10] Zontone P., Affanni A., Bernardini R., Piras A., Rinaldo R. Stress detection through electrodermal activity (EDA) and electrocardiogram (ECG) analysis in car drivers. Proceedings of the 27th European Signal Processing Conference (EUSIPCO).

[11] Manzalini A. (2019). Towards a Quantum Field Theory for Optical Artificial Intelligence. Ann. Emerg. Technol. Comput. (AETiC) Print ISSN.

[12] Sánchez-Sánchez C., Izzo D., Hennes D. Learning the optimal state-feedback using deep networks. Proceedings of the IEEE Symposium Series on Computational Intelligence (SSCI).

[13] LeCun Y., Bengio Y., Hinton G. (2015). Deep Learning. Nature.

[14] Mukhopadhyay A.K., Samui S. (2020). An experimental study on upper limb position invariant EMG signal classification based on deep neural network. Biomed. Signal Process. Control.

[15] Tajbakhsh N., Shin J.Y., Gurudu S.R., Hurst R.T., Kendall C.B., Gotway M.B., Liang J. (2016). Convolutional neural networks for medical image analysis: Full training or fine tuning?. IEEE Trans. Med. Imaging.

[16] Paul R., Hawkins S.H., Balagurunathan Y., Schabath M.B., Gillies R.J., Hall L.O., Goldgof D.B. (2016). Deep feature transfer learning in combination with traditional features predicts survival among patients with lung adenocarcinoma. Tomography.

[17] Hatt M., Tixier F., Visvikis D., Cheze Le Rest C. (2017). Radiomics in PET/CT: More than meets the eye?. J. Nucl. Med..

[18] Bizzego A., Bussola N., Chierici M., Maggio V., Francescatto M., Cima L., Cristoforetti M., Jurman G., Furlanello C. (2019). Evaluating reproducibility of AI algorithms in digital pathology with DAPPER. PLoS Comput. Biol..

[19] Bizzego A., Bussola N., Salvalai D., Chierici M., Maggio V., Jurman G., Furlanello C. Integrating deep and radiomics features in cancer bioimaging. Proceedings of the IEEE Conference on Computational Intelligence in Bioinformatics and Computational Biology (CIBCB).

[20] Tseng H.H., Wei L., Cui S., Luo Y., Ten Haken R.K., El Naqa I. (2018). Machine learning and imaging informatics in Oncology. Oncology.

[21] Topol E.J. (2019). High-performance medicine: The convergence of human and artificial intelligence. Nat. Med..

[22] Esteva A., Kuprel B., Novoa R.A., Ko J., Swetter S.M., Blau H.M., Thrun S. (2017). Dermatologist-level classification of skin cancer with deep neural networks. Nature.

[23] Mobadersany P., Yousefi S., Amgad M., Gutman D.A., Barnholtz-Sloan J.S., Velázquez Vega J.E., Brat D.J., Cooper L.A.D. (2018). Predicting cancer outcomes from histology and genomics using convolutional networks. Proc. Natl. Acad. Sci. USA.

[24] Wieclaw L., Khoma Y., Fałat P., Sabodashko D., Herasymenko V. Biometrie identification from raw ECG signal using deep learning techniques. Proceedings of the 9th IEEE International Conference on Intelligent Data Acquisition and Advanced Computing Systems: Technology and Applications (IDAACS).

[25] Mathews S.M., Kambhamettu C., Barner K.E. (2018). A novel application of deep learning for single-lead ECG classification. Comput. Biol. Med..

[26] Xu S.S., Mak M.W., Cheung C.C. (2018). Towards end-to-end ECG classification with raw signal extraction and deep neural networks. IEEE J. Biomed. Health Inform..

[27] Yu D., Sun S. (2020). A systematic exploration of deep neural networks for EDA-based emotion recognition. Information.

[28] Rim B., Sung N.J., Min S., Hong M. (2020). Deep Learning in physiological signal data: A survey. Sensors.

[29] Yildirim O., Baloglu U.B., Tan R.S., Ciaccio E.J., Acharya U.R. (2019). A new approach for arrhythmia classification using deep coded features and LSTM networks. Comput. Methods Programs Biomed..

[30] Oh S.L., Ng E.Y., San Tan R., Acharya U.R. (2018). Automated diagnosis of arrhythmia using combination of CNN and LSTM techniques with variable length heart beats. Comput. Biol. Med..

[31] Ahmedt-Aristizabal D., Fookes C., Denman S., Nguyen K., Sridharan S., Dionisio S. (2019). Aberrant epileptic seizure identification: A computer vision perspective. Seizure.

[32] Mumtaz W., Qayyum A. (2019). A deep learning framework for automatic diagnosis of unipolar depression. Int. J. Med. Inform..

[33] Golmohammadi M., Harati Nejad Torbati A.H., Lopez de Diego S., Obeid I., Picone J. (2019). Automatic analysis of EEGs using big data and hybrid deep learning architectures. Front. Hum. Neurosci..

[34] Chambon S., Galtier M.N., Arnal P.J., Wainrib G., Gramfort A. (2018). A deep learning architecture for temporal sleep stage classification using multivariate and multimodal time series. IEEE Trans. Neural Syst. Rehabil. Eng..

[35] Andreotti F., Phan H., Cooray N., Lo C., Hu M.T., De Vos M. Multichannel sleep stage classification and transfer learning using convolutional neural networks. Proceedings of the 40th Annual International Conference of the IEEE Engineering in Medicine and Biology Society (EMBC).

[36] Allen J. (2007). Photoplethysmography and its application in clinical physiological measurement. Physiol. Meas..

[37] Benedek M., Kaernbach C. (2010). A continuous measure of phasic electrodermal activity. J. Neurosci. Methods.

[38] Taylor S., Jaques N., Chen W., Fedor S., Sano A., Picard R. Automatic identification of artifacts in electrodermal activity data. Proceedings of the 37th Annual International Conference of the IEEE Engineering in Medicine and Biology Society (EMBC).

[39] Künecke J., Hildebrandt A., Recio G., Sommer W., Wilhelm O. (2014). Facial EMG responses to emotional expressions are related to emotion perception ability. PLoS ONE.

[40] Mavratzakis A., Herbert C., Walla P. (2016). Emotional facial expressions evoke faster orienting responses, but weaker emotional responses at neural and behavioural levels compared to scenes: A simultaneous EEG and facial EMG study. Neuroimage.

[41] Lundqvist L.O. (1995). Facial EMG reactions to facial expressions: A case of facial emotional contagion?. Scand. J. Psychol..

[42] Urfy M.Z., Suarez J.I. (2014). Breathing and the nervous system. Handbook of Clinical Neurology.

[43] Albinali F., Goodwin M.S., Intille S.S. Recognizing stereotypical motor movements in the laboratory and classroom: A case study with children on the autism spectrum. Proceedings of the 11th International Conference on Ubiquitous Computing.

[44] Pan C.Y., Tsai C.L., Hsieh K.W., Chu C.H., Li Y.L., Huang S.T. (2011). Accelerometer-determined physical activity among elementary school-aged children with autism spectrum disorders in Taiwan. Res. Autism Spectr. Disord..

[45] Memari A., Ghaheri B., Ziaee V., Kordi R., Hafizi S., Moshayedi P. (2013). Physical activity in children and adolescents with autism assessed by triaxial accelerometry. Pediatr. Obes..

[46] Koelstra S., Muhl C., Soleymani M., Lee J.S., Yazdani A., Ebrahimi T., Pun T., Nijholt A., Patras I. (2011). DEAP: A database for emotion analysis; Using physiological signals. IEEE Trans. Affect. Comput..

[47] Bizzego A., Gabrieli G., Furlanello C., Esposito G. (2020). Comparison of wearable and clinical devices for acquisition of peripheral nervous system signals. Sensors.

[48] Bizzego A., Azhari A., Campostrini N., Truzzi A., Ng L.Y., Gabrieli G., Bornstein M.H., Setoh P., Esposito G. (2020). Strangers, friends, and lovers show different physiological synchrony in different emotional states. Behav. Sci..

[49] Gabrieli G., Bornstein M.H., Esposito G. (2019). Using users’ physiological responses for the estimation of websites’ aesthetic judgments. PsyArXiv.

[50] Da Silva H.P., Guerreiro J., Lourenço A., Fred A.L., Martins R. (2014). BITalino: A novel hardware framework for physiological computing. PhyCS.

[51] Shorten C., Khoshgoftaar T.M. (2019). A survey on image data augmentation for deep learning. J. Big Data.

[52] Ioffe S., Szegedy C. (2015). Batch normalization: Accelerating deep network training by reducing internal covariate shift. arXiv.

[53] Shang W., Sohn K., Almeida D., Lee H. Understanding and improving convolutional neural networks via concatenated rectified linear units. Proceedings of the International Conference on Machine Learning.

[54] Hochreiter S., Schmidhuber J. (1997). Long Short-Term Memory. Neural Comput..

[55] Graves A., Mohamed A.r., Hinton G. Speech recognition with deep recurrent neural networks. Proceedings of the IEEE International Conference on Acoustics, Speech and Signal Processing.

[56] Zhang Z., Sabuncu M. (2018). Generalized cross entropy loss for training deep neural networks with noisy labels. Adv. Neural Inf. Process. Syst..

[57] Kingma D.P., Ba J. (2014). Adam: A method for stochastic optimization. arXiv.

[58] Jurman G., Riccadonna S., Furlanello C. (2012). A comparison of MCC and CEN error measures in multi-class prediction. PLoS ONE.

[59] Pedregosa F., Varoquaux G., Gramfort A., Michel V., Thirion B., Grisel O., Blondel M., Prettenhofer P., Weiss R., Dubourg V. (2011). Scikit-learn: Machine Learning in Python. J. Mach. Learn. Res..

[60] Paszke A., Gross S., Massa F., Lerer A., Bradbury J., Chanan G., Killeen T., Lin Z., Gimelshein N., Antiga L., Wallach H., Larochelle H., Beygelzimer A., d’Alché-Buc F., Fox E., Garnett R. (2019). PyTorch: An imperative style, high-performance Deep Learning library. Advances in Neural Information Processing Systems 32.

[61] Dhaouadi S., Ben Khelifa M.M. A multimodal physiological-based stress recognition: Deep Learning models’ evaluation in gamers’ monitoring application. Proceedings of the 5th International Conference on Advanced Technologies for Signal and Image Processing (ATSIP).

[62] Shu L., Xie J., Yang M., Li Z., Li Z., Liao D., Xu X., Yang X. (2018). A review of emotion recognition using physiological signals. Sensors.

[63] Rasheed K., Qayyum A., Qadir J., Sivathamboo S., Kwan P., Kuhlmann L., O’Brien T., Razi A. (2020). Machine learning for predicting epileptic seizures using EEG signals: A review. IEEE Rev. Biomed. Eng..

[64] Sun W., Zheng B., Qian W. (2017). Automatic feature learning using multichannel ROI based on deep structured algorithms for computerized lung cancer diagnosis. Comput. Biol. Med..

[65] Li Z., Wang Y., Yu J., Guo Y., Cao W. (2017). Deep learning based radiomics (DLR) and its usage in noninvasive IDH1 prediction for low grade glioma. Sci. Rep..

[66] Kontos D., Summers R.M. (2018). Radiomics and Deep Learning. J. Med. Imaging.

[67] Arimura H., Soufi M., Kamezawa H., Ninomiya K., Yamada M. (2019). Radiomics with artificial intelligence for precision medicine in radiation therapy. J. Radiat. Res..

[68] Azhari A., Lim M., Bizzego A., Gabrieli G., Bornstein M.H., Esposito G. (2020). Physical presence of spouse enhances brain-to-brain synchrony in co-parenting couples. Sci. Rep..

[69] Azhari A., Gabrieli G., Bizzego A., Bornstein M.H., Esposito G. (2020). Probing the association between maternal anxious attachment style and mother-child brain-to-brain coupling during passive co-viewing of visual stimuli. Attach. Hum. Dev..

[70] Simonyan K., Zisserman A. (2014). Very deep convolutional networks for large-scale image recognition. arXiv.

